# The Global Trends of Thyroid Cancer Research: A Scientometric Study

**DOI:** 10.1155/2024/5747618

**Published:** 2024-08-26

**Authors:** Morteza Ghojazadeh, Majid Mobasseri, Hadi Mostafaei, Mahsa Asadizadeh-Azar, Neda Kabiri, Abdolhassan Kazemi, Alireza Lotfi, Reza Aletaha, Ali Akbari-Khoei, Hanieh Salehi-Pourmehr

**Affiliations:** ^1^ Neurosciences Research Center Tabriz University of Medical Sciences, Tabriz, Iran; ^2^ Research Center for Evidence-Based Medicine Iranian EBM Centre: A JBI Centre of Excellence Faculty of Medicine Tabriz University of Medical Sciences, Tabriz, Iran; ^3^ Endocrinology Research Center Tabriz University of Medical Sciences, Tabriz, Iran; ^4^ Department of Urology Comprehensive Cancer Center Medical University of Vienna, Vienna, Austria; ^5^ Student Research Committee Tabriz University of Medical Sciences, Tabriz, Iran; ^6^ Medical Philosophy and History Research Center Tabriz University of Medical Sciences, Tabriz, Iran

**Keywords:** bibliometrics, scientometry, Scopus, thyroid cancer

## Abstract

Cancer of the thyroid has become the fastest-growing cancer among women in the past several decades. This study is aimed at using scientometric methods to identify research frontiers and development trends in the field of thyroid cancer (TC) research. We used the Scopus database to collect articles and reviews related to TC in November 2022. R software and Bibliometrix software package were used for scientometric analysis. More than 28,000 articles were obtained from Scopus using the defined specific keywords. The United States, France, and England published the most publications. Journal of Clinical Endocrinology and Metabolism and Cancer were found as the core journals in this field. Morris LGT, Sikora AG, and Davies L authored the most publications. National Cancer Institute, National Cancer Institute at NIH, and the University of Washington contributed the most publications. The most cited evidence was related to the articles of Bray (2018), Sung (2021), and Parkin (2005). Using scientometric analysis, this study mapped and visualized the knowledge landscape in the field of TC. The analysis showed that scientists in the field of TC are working collaboratively to tackle one of the most prevalent cancers in the world. This analysis showed that scientists in the field of TC are working with a scientific framework as a team to tackle one of the most common cancers in the world.

## 1. Introduction

Thyroid carcinoma or thyroid cancer (TC), a malignant neoplasm of the thyroid gland, is the most prevalent malignancy of the endocrine system [1]. Some of the main risk factors for this cancer are radiation exposure and being overweight or obese [2]. Cancer of the thyroid has become the fastest-growing cancer among women in the past several decades [3]. The Global Cancer Statistics (GLOBOCAN) reported that there were 586,202 new cases of TC and 43,646 new deaths in 2020 in the world [4]. The growing research in the field of TC plays an important role in guiding recommendations in diagnosis and treatment, as well as generating hypotheses about changing agents of incidence and mortality trends in this field [5–8].

Bibliometrics is the science of analysis of published resources using quantitative and statistical techniques to indicate the status and relationship between published work [9]. Bibliometric research is generally composed of two main analytical techniques including assessment of productivity and impact and science mapping [10]. Some aspects of TC have been analyzed in scientometric studies in recent decades. As an example, research conducted on scientometric analysis of TC in the years 2010–2015 using the Web of Science database showed the growing number of publications in this field [11]. Also, Raja, Ramkumar, and Viji, in their scientometric study, assessed the gender dimension in TC during 1991–2010 [12]. The effect of selenium in TC was assessed in a scientometric study conducted by Pakdel et al. in 2019, indicating intersubject relationships present in the literature in this field [13]. Despite previous scientometric works on specific aspects of this topic, no comprehensive scientometric analysis has been conducted on the global trends of TC research. There is a gap in a broad overview of the current research situation on TC. We conducted the current scientometric analysis to characterize the current research in the field of TC. We aimed to obtain information about the most representative countries and institutions, journals, authors, and keywords in TC publications in order to provide direction for future researchers.

## 2. Materials and Methods

### 2.1. Search Strategy

Without language or date restrictions, we searched for TC research using the Scopus database in November 2022. We included publication types of systematic reviews and meta-analysis, narrative reviews, case reports, and case series from the first published to the present. Nonoriginal articles were excluded. Search terms included TITLE-ABS-KEY (Thyroid AND (Cancer OR malignancy OR neoplasm OR Carcinoma)). These terms were chosen in accordance with Medical Subject Heading (Mesh). We searched for our keywords in the title of articles in order to ensure that the relevant articles to TC research were included in the analysis. We did not search the abstract, introduction, or any other article section. Only English journals and articles were included in the analysis. The retrieved results were screened based on the title and abstract. Full texts were screened by two of the authors for inclusion criteria.

### 2.2. Analysis

The online Scopus analysis tool was applied to record the identified articles and citations and to gain information about countries, authors, institutions, and journals. We conducted the complete scientometric analysis using Bibliometrix, an R tool of R-studio (version 4.2.1), and visualized the results with Bibliometrix images.

## 3. Results and Discussion

### 3.1. Annual Growth Trend

More than 28,000 papers related to this subject were retrieved from the Scopus database, of which, 4288 belong to the last 6 years (2017–2022). The average annual output of the last 6 years was 714 publications per year. The peak annual output of the TC was in 2021 (*n* = 933).  Table 1 shows the annual output of TC from 2017 to 2022.

### 3.2. Countries and Institutions

The Top 10 countries that participated in TC research are shown in Table 2. Remarkably, most of the studies related to the topic were published in the United States (*n* = 435706), followed by France (*n* = 118949), and the United Kingdom (*n* = 57734). Six of the Top 10 countries belong to Europe. Two of the remaining belong to Asia (Japan and Korea). The remaining two countries were the United States and Canada.

**Figure 1:**
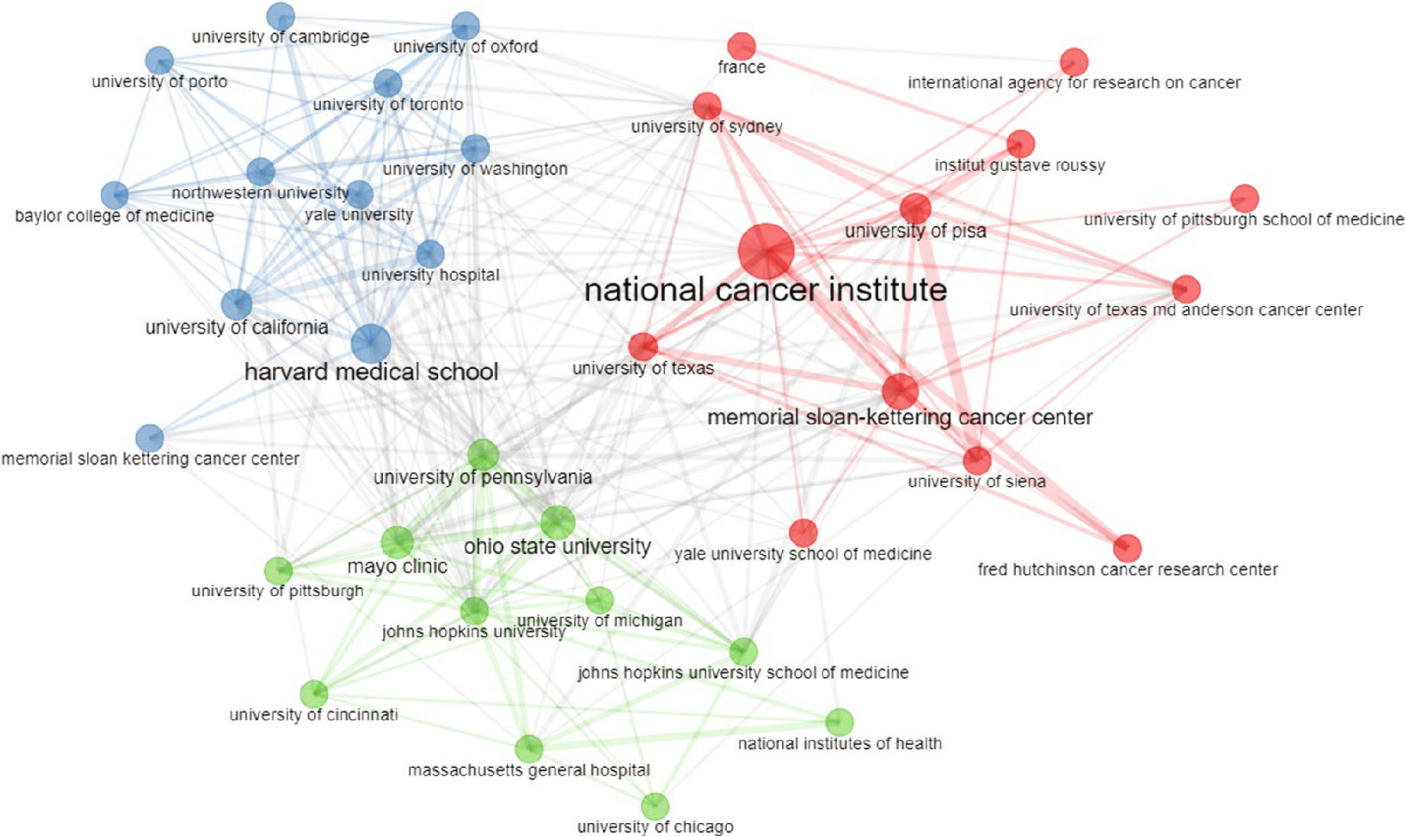
The network map of institutions participating in thyroid cancer research.

Analysis of the 2000 most-cited publications showed that among the contributing institutions, the National Cancer Institute ranked first (*n* = 349), followed by the National Cancer Institute at NIH (*n* = 243), and the University of Washington (*n* = 241) (Figure 1).

### 3.3. Journals and Cocited Journals

**Figure 2:**
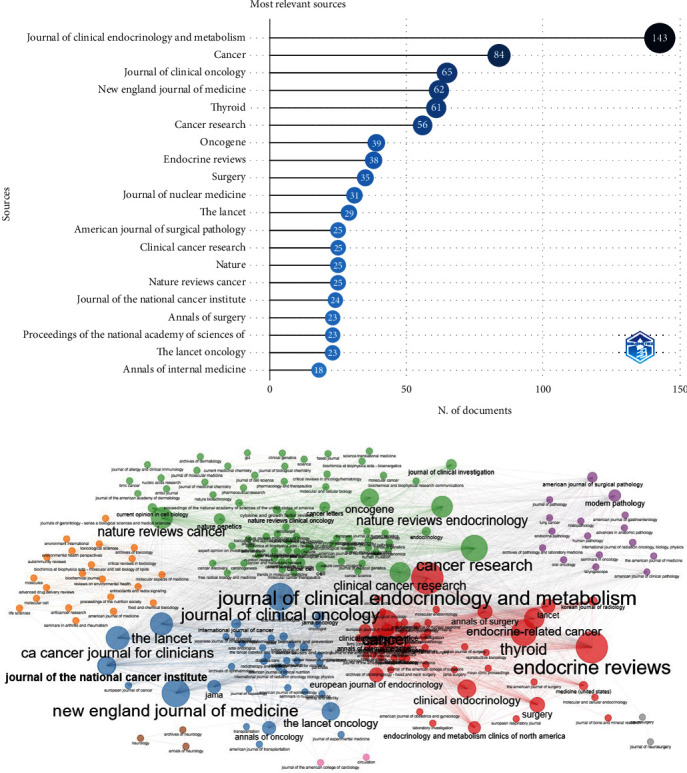
(a) The Top 20 journals collaborated in the field of thyroid cancer. (b) The network map of academic journals and cocited academic journals for thyroid cancer research.

The Top 10 academic journals involved in TC research are shown in Table 3. Moreover, the Top 20 productive journals in TC are shown in Figure 2(a). Among them, the *Journal of Clinical Endocrinology and Metabolism* (*n* = 143, IF (impact factor) 2021 = 6.134, Q1) ranked first, followed by *Cancer* (*n* = 84, IF 2021 = 6.921, Q1), and the *Journal of Clinical Oncology* (*n* = 65, IF 2021 = 50.739, Q1). All of these three are published in the United States. The network map of academic journals and their cocitations is presented in Figure 2(b).

### 3.4. Authors and Cocited Authors

**Figure 3:**
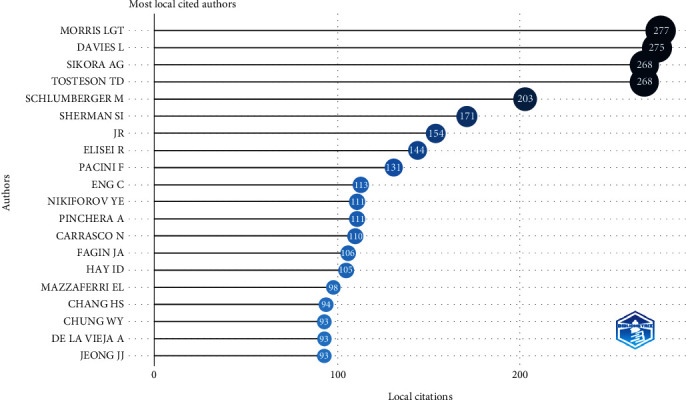
The Top 20 author's production in the field of thyroid cancer.

**Figure 4:**
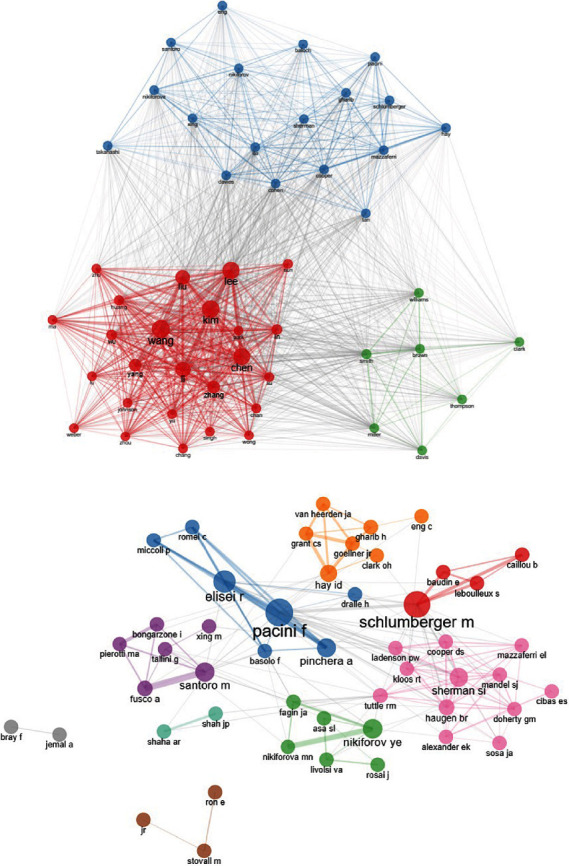
(a) The cocitation analysis of authors. (b) Author collaboration network.

The Top 20 author's production in this field is shown in Figure 3. Morris LGT was the most contributing author in this field with 277 published articles, followed by Davis L with 275 publications and Sikora AG and Tosteson TD both with 268 articles. The analysis of the cocitation of authors and author collaboration network are indicated in Figures 4(a) and 4(b), respectively. The size of the nodes indicates the number of publications, while the thickness of the links shows the intensity of the collaborations.

### 3.5. Coword Evaluation

**Figure 5:**
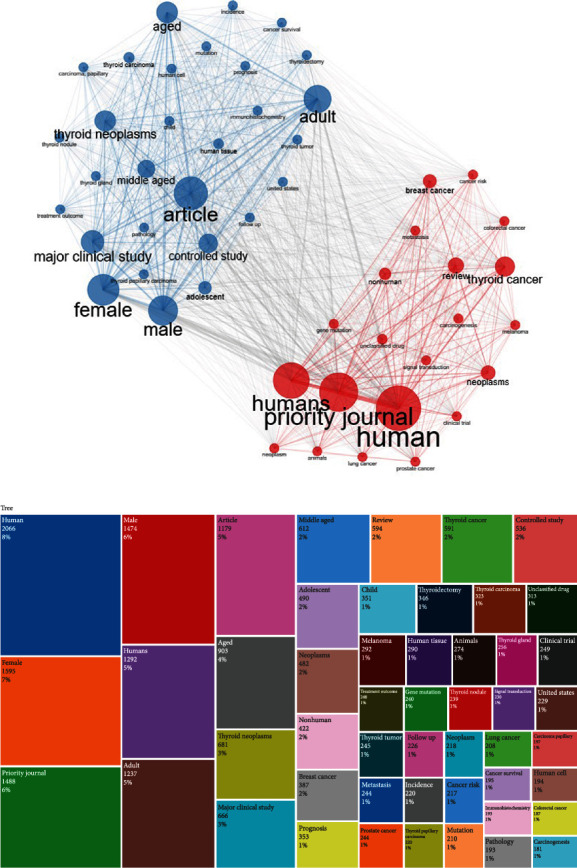
(a) Cooccurring words network. (b) Keyword tree map.

Coword analysis indicates the cooccurrence of keywords in the researched topics, as well as the interaction among the searched keywords. Figures 5(a) and 5(b) show the cooccurring words network and keyword tree map. The most frequently occurring words included human, female, priority journal, male, humans, adult, and article.

## 4. Discussion

A scientometric analysis of TC publication output was conducted in the current study. There were more than 28,000 publications in this field. The major aim of the current analysis was to visualize and analyze the status of countries, organizations, and authors that played important roles in the field of TC research. Analyzing these publications can be beneficial in enlightening the research path of the field with respect to countries and institutions, journals, authors, and word evaluations.

The most productive countries were the United States (*n* = 435,706), France (*n* = 118,949), and the United Kingdom (*n* = 57,734). Among the Top 10 productive countries, only two were from Asia. It is apparent that there is inequity in TC research production between the developing and developed world. Other scientometric analyses in different fields indicated that the United States was the leading country with the maximum number of publications [14–18]. On the contrary, based on research by Li et al., China was the most contributing country in the field of autoimmune thyroiditis research [19]. This inequity indicates a strong need for improving international collaboration among authors.

Among the affiliations that contributed to this field, the National Cancer Institute, the National Cancer Institute at NIH, and the University of Washington were the Top 3, all from the United States. Only one of the Top 10 institutions collaborated in this field belongs to Europe (Italy), and the remaining are all from the United States. Similarly, Xing et al. [20] showed in their analysis that the National Cancer Institute was the top research institution in the field of cervical cancer. Also, based on a scientometric analysis conducted by Ruiz-Coronel, Andrade, and Carrillo-Calvet [21], the National Cancer Institute ranked fourth among the 10 Mexican public health institutions indexed in the Web of Science. Memorial Sloan Kettering Cancer Center was the second most productive organization in the field of breast cancer [22]. This organization was similarly among the Top 10 organizations contributing to the TC research in the current study.

The Top 10 journals in this field published 614 papers, all from the United States and the United Kingdom (*n* = 1). All were developed countries. All of the Top 10 journals are among the Quartile 1 (Q1) ranking, indicating that high-impact journals contribute more to the field of TC. The highest impact factor belongs to The *New England Journal of Medicine* (IF = 176.82) with 62 published papers in the field. *Journal of Clinical Endocrinology and Metabolism* was the top contributing journal based on our analysis. The journal was the third one in the field of Selenium effect on thyroid disorders based on a scientometric analysis by Pakdel et al. [13]. Based on the results of a scientometric analysis by Biglu Abotalebi, and Ghavami [22], the *Journal of Cancer Research* was the most productive journal in the field of breast cancer during 2006–2015, which was followed by the *Journal of Clinical Oncology*. Similarly, these were among the Top 10 journals in TC based on the analysis from the current research.

### 4.1. Strengths and Limitations

A scientometric analysis provides a visual illustration of evolving research in the field, which is highly comprehensive and objective when compared to standard reviews. However, our study has some limitations. A recent similar study was published in 2023 by Song et al. [23], assessing scientific trends on all thyroid disorders, found that the United States followed by China and Italy had the highest publication volume and citations. Also, *Journals of Thyroid*, *Journal of Clinical Endocrinology & Metabolism,* and *Clinical Endocrinology* were the most prolific journals in this field of thyroid disorders. Although this study and our study have the same search date, which was November 2022, in our study, we searched the Scopus database from the first published, while in the study by Song and colleagues, they searched the Web of Science database from January 2000. Also, we specifically assessed TC which is the most common among thyroid disorders. The major limitation of this study was the only database search of SCOPUS; however, this database collects information that exactly reflects the field, since a considerable percentage of indexed high-impact factor journals. Previous studies have demonstrated that because of the high percentage of indexed high-impact-factor journals in the Scopus database, studies restricted to this database appear to collect datasets that accurately reflect fields. The largest abstract and citation database for peer-reviewed literature, including books, scientific journals, and conference proceedings, is called Scopus. Scopus offers intelligent tools for tracking, analyzing, and visualizing research and provides a thorough picture of the global research output in the domains of science, technology, medicine, social sciences, and arts and humanities. The other reason why we used this database was that we had institutional access to Scopus, and it offers a wider coverage and output as compared to PubMed. One other limitation of this study was that we excluded non-English documents; thus, papers in other languages may be missed. In addition, study-type restriction was the other limitation of this study, since we excluded nonoriginal articles.

## 5. Conclusion

The analysis of the literature related to TC shows the historical development, the core of the research clusters, and the relationships between them with incremental publication activity in the last decades. Citation explosions reveal emerging trends and stakeholders. The analysis of the Top 2000 subcategories or citations provides insight into original articles, highly cited topics, types of studies, and international leaders of the research field. This provides an opportunity for future researchers to exploit the characteristics of TC to generate higher-quality evidence.

## Figures and Tables

**Table 1:** Annual output reports of thyroid cancer from 2017 to 2022.

**Year**	**Articles**
2017	5025
2018	5052
2019	5519
2020	6386
2021	7609
2022	7530

**Table 2:** The Top 10 countries and institutions involved in thyroid cancer research.

**Rank**	**Counties**	**Count**	**Institution**	**Count**
1	USA	435706	National Cancer Institute	349
2	France	118949	National Cancer Institute At NIH	243
3	United Kingdom	57734	University Of Washington	241
4	Italy	54730	Memorial Sloan-Kettering Cancer Center	216
5	Japan	21978	Harvard Medical School	214
6	Germany	21345	University of Pisa	192
7	Canada	20219	University of California	175
8	Netherlands	15826	Mayo Clinic	159
9	Belgium	13253	Ohio State University	142
10	Korea	12710	Institute for Health Metrics and Evaluation	112

**Table 3:** The Top 10 academic journals involved in thyroid cancer research.

**Rank**	**Journals**	**Count**
1	Journal of Clinical Endocrinology and Metabolism	143
2	Cancer	84
3	Journal of Clinical Oncology	65
4	New England Journal of Medicine	62
5	Thyroid	61
6	Cancer Research	56
7	Oncogene	39
8	Endocrine Reviews	38
9	Surgery	35
10	Journal of Nuclear Medicine	31

## Data Availability

The data that support the findings of this study are available from the corresponding author upon reasonable request.
